# Contribution of the peroneus longus neuromuscular compartments to eversion and plantarflexion of the ankle

**DOI:** 10.1371/journal.pone.0250159

**Published:** 2021-04-15

**Authors:** Guillermo Mendez-Rebolledo, Rodrigo Guzmán-Venegas, Oscar Valencia, Kohei Watanabe

**Affiliations:** 1 Escuela de Kinesiología, Facultad de Salud, Universidad Santo Tomás, Santiago, Chile; 2 Laboratorio Integrativo de Biomecánica y Fisiología del Esfuerzo (LIBFE), Escuela de Kinesiología, Facultad de Medicina, Universidad de los Andes, Santiago, Chile; 3 Laboratory of Neuromuscular Biomechanics, Faculty of Liberal Arts and Sciences and School of International Liberal Studies, Chukyo University, Nagoya, Japan; National Tsing Hua University, TAIWAN

## Abstract

Compartmentalization of animal and human skeletal muscle by multiple motor nerve branches known as the neuromuscular compartment (NMC) has been observed primarily in muscles that participate in a plane of motion. In this context, the peroneus longus muscle contributes to eversion and plantarflexion of the ankle and the presence of NMCs has been reported. However, no research has reported the selective activation of the compartments of the peroneus longus during the performance of different ankle movements. The purpose of this research was to determine the contribution of peroneus longus NMCs, through multi-channel surface electromyography (sEMG), to eversion and plantarflexion movements. Multi-channel sEMG was recorded from the peroneus longus muscle by using an electrode grid during eversion and plantarflexion of the ankle at 10%, 30%, 50%, and 70% of maximal voluntary isometric contraction (MVIC). The root mean square and displacement of the center of mass position in the X (COMx) and Y (COMy) components were calculated. The primary finding was that eversion showed significantly higher sEMG amplitude than plantarflexion in the posterior compartment in low, moderate, and high percentages of MVIC. However, no significant difference in sEMG amplitude was observed in the anterior compartment between eversion and plantarflexion. In addition, a posterior displacement of the COMx in eversion compared to plantarflexion in all MVIC percentages, with greater topographic distancing of the COMx at higher levels of activation. In conclusion, the peroneus longus muscle presented NMCs; the anterior compartment contributed to both eversion and plantarflexion movements, whereas the posterior compartment mainly contributed to the eversion movement of the ankle in low, moderate, and high percentages of MVIC.

## Introduction

Compartmentalization of animal and human skeletal muscles by multiple motor nerve branches, known as the neuromuscular compartment (NMC), has been observed primarily in muscles that participate in a plane of motion [1–3]. These compartments present a spatial distribution depending on the motor task [2–5] and have been investigated through anatomical-cadaveric [6, 7], physiological-electromyographic [3, 4, 8, 9], and biomechanical studies [5, 9–11] in the trapezius [12], extensor digitorum communis [13], rectus femoris [3, 9, 11], hamstring [14], medial gastrocnemius [15], and masseter muscles [4], among others. Previous reports demonstrated region-specific activation properties of bi-articular muscles by using multi-channel surface electromyography (sEMG) [3, 14]. For example, proximal and distal regions of the rectus femoris had higher sEMG amplitude during hip flexion and knee extension, respectively [3]. These investigations focused on mono or bi-articular muscles that have clearly defined functions in a plane of movement. However, there are few reports on neuromuscular compartmentalization in muscles that participate in two or more planes of movement. In this context, the peroneus longus muscle presents a compartmental subdivision from an anatomical and biomechanical point of view [5–7], and this muscle contributes to eversion and plantarflexion during motor tasks like walking and running on inclined planes [16–18].

The peroneus longus has a muscle belly with an average length of 12.4 ± 1.91 cm [19] and a compartmental anatomical organization [6, 7, 20]. This architectural subdivision has been reported in anatomical studies where four compartments have been observed, two deep and two superficial, organized around a central connective tissue with a longitudinal axis [6]. The superficial compartments are called anterior and posterior, and they can be separately recorded by sEMG [6, 7]. These characteristics have allowed the recording of its electrical activity through multi-channel sEMG using a 4 x 3 electrode grid and a 20 mm interelectrode distance [21]. This subdivision has been reported in biomechanical studies, in which through the differentiated stimulation of the peroneus longus compartments, it was observed that the posterior compartment contributes mainly to eversion of the ankle, and the anterior compartment contributes mainly to dorsiflexion of the foot in a non-weight bearing position [5]. However, no research has reported the muscle compartmentalization during a voluntary motor task, i.e., the selective activation of the compartments of the peroneus longus during the performance of different ankle movements. For these reasons, it is clinically relevant to identify the compartmentalization of the peroneus longus muscle during the execution of the movements of eversion and plantarflexion of the ankle. Their knowledge would allow updating the models of ankle joint dysfunction (e.g., chronic ankle instability); as well as physical therapy, therapeutic exercise and neuromuscular electrostimulation, which until now only consider the peroneus longus muscle as a single neuromuscular unit [22–24].

Multi-channel sEMG has been used on the masseter muscles [4], rectus femoris [3, 9] and medial gastrocnemius [15] to record the electrical activity of the motor units that are part of the various NMCs. This technique is based on the location of electrodes in a two-dimensional matrix allowing the muscle’s electrical activity of a broad recording area to be obtained, unlike single-channel and fine-wire electromyography, which only allow obtaining the electrical activity of a small area of the skeletal muscle [8]. In this context, the selective activation of the compartments may result in changes in spatial distribution of sEMG, and the use of multi-channel sEMG may be useful to describe these changes. In addition, the use of multi-channel sEMG can facilitate the recognition of peroneus longus NMCs during the execution of movements and motor tasks and thus contribute to the design and understanding of therapeutic exercises for the rehabilitation processes of musculoskeletal and neurological dysfunctions. For this, multi-channel sEMG uses the maximum voluntary isometric contraction (MVIC) method to obtain a normalized amplitude of activation for each NMC. Furthermore, by calculating and recognizing the position of the center of mass (COM), changes in a muscle’s spatial distribution can be evidenced at different levels of contraction [4, 25].

As stated above, the purpose of this research was to determine the contribution of peroneus longus NMCs, through multi-channel sEMG, to eversion and plantarflexion movements of the ankle. We hypothesize that the peroneus longus muscle presents NMCs compatible with a spatial distribution depending on the motor task; specifically, the anterior compartment contributes to plantarflexion, whereas the posterior compartment mainly contributes to eversion movement of the ankle.

## Materials and methods

### Participants

All procedures were carried out in accordance with the Code of Ethics of the World Medical Association (Declaration of Helsinki) for experiments on humans and the STROBE statement [26]. Participants were selected through a non-probability sample of a group of students (male) from the Faculty of Medicine of the Universidad de los Andes, Chile. The research participants read and signed an informed consent evaluated and approved by the Scientific Ethics Committee of the Universidad de los Andes, Chile (CEC201838).

Accepting an alpha risk of 0.05, a beta risk of 0.2 in a bilateral contrast and a standard deviation of 11%, twenty-two male participants were required to detect a difference equal to or greater than 7% at 60% MIVC. Although there are no studies evaluating the sEMG activity of the peroneus longus muscle compartments, the mean and standard deviation of the root mean square (RMS) at 60% of the MIVC were used. These criteria were based on previous studies where statistically significant differences were observed between compartments of a muscle [3, 4].

**Fig 1:**
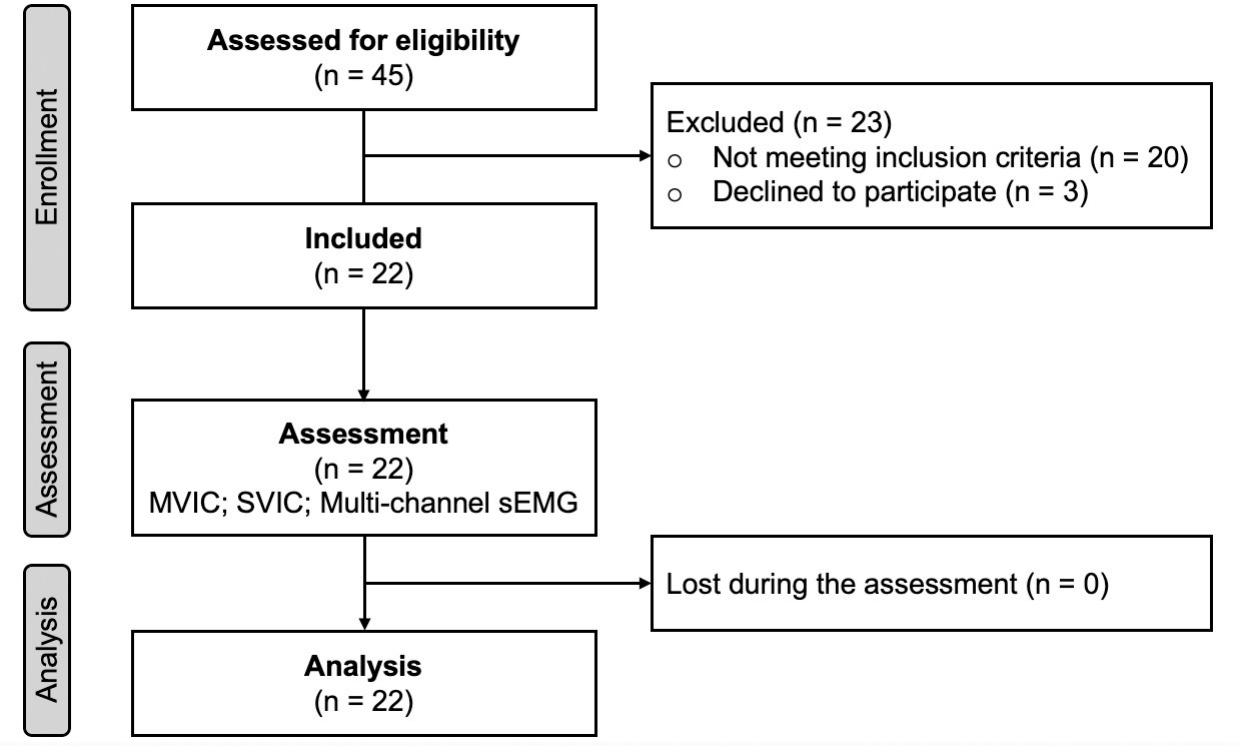
Flowchart about the study design. MVIC, maximum voluntary isometric contraction; SVIC, submaximal voluntary isometric contraction; sEMG, surface electromyography.

Participants presented the following inclusion criteria: (i) age between 18 and 40 years, since it has been observed that the morphology and neuromuscular activation of human skeletal muscle are regionally affected by older ages (aging) [27]; (ii) moderate physical activity level of 30 min per session at least three times a week [28]; (iii) twenty-two or more points in the Functional Assessment Tool of the Ankle Joint, which indicates that the individual does not present signs of functional instability of the ankle joint [29]; and (iv) reading and signing an informed consent. Participants were excluded if they presented: (i) history of two or more lateral ankle sprains; (ii) pain, instability, or ankle weakness at the time of the evaluation; and (iii) history of any other injury to the lower limb in the last six months prior to the evaluation [29]. Finally, twenty-two volunteers (age = 24.9 ± 4.9 years old; body mass = 75.1 ± 5.7 kg; height = 174.7 ± 4.2 cm) were selected. **Fig 1** shows a flowchart of the enrollment process of the participants.

### Experimental design

This research presented a cross-sectional design in which a group of participants was evaluated in one session (**Fig 1**). The dominant lower limb was used for all the research procedures. The dominant lower limb was determined as the limb the subject used to kick a ball. The participants performed MVIC and submaximal isometric voluntary contractions during plantarflexion and eversion ankle movements. During submaximal contractions, multi-channel sEMG (EMG-USB2, OT Biolettronica, Torino, Italy) was recorded from the peroneus muscle. Both movements were performed following the submaximal (10%, 30%, 50%, and 70%) isometric voluntary contraction test.

#### Maximum voluntary isometric contraction recording

Isometric plantarflexion and eversion joint torque were performed on a custom ankle brace dynamometer (ST-1, Kinetecnics, Santiago, Chile) mounting a torque sensor (TRS-2 K, Transducer Techniques LLC, USA). For this, participants sat in a chair with the back fully against the backrest of it. The hip was set at 90° flexion. Each volunteer was asked to keep their arms crossed over their chest. The dominant leg and foot were placed in the custom ankle brace dynamometer to fix the ankle joint in a neutral position, taking care to keep the knee in 60° flexion. Two torque sensors into the aluminum frame were adjusted to coincide with the talotibial and subtalar joint axes for plantarflexion and eversion movements of the ankle, respectively. This procedure was designed respecting a similar setup observed in previous research on MVIC of the ankle muscles [30]. All participants were asked to perform three maximum isometric contractions against the sensor to each plantarflexion and eversion movement, with vigorous encouragement from the investigators when the force began to plateau. The maximum isometric contraction lasted five seconds and the rest period between trials was one minute. The magnitude of the MVIC was defined as the maximum force value recorded in the three trials.

#### Multi-channel sEMG recording

**Fig 2:**
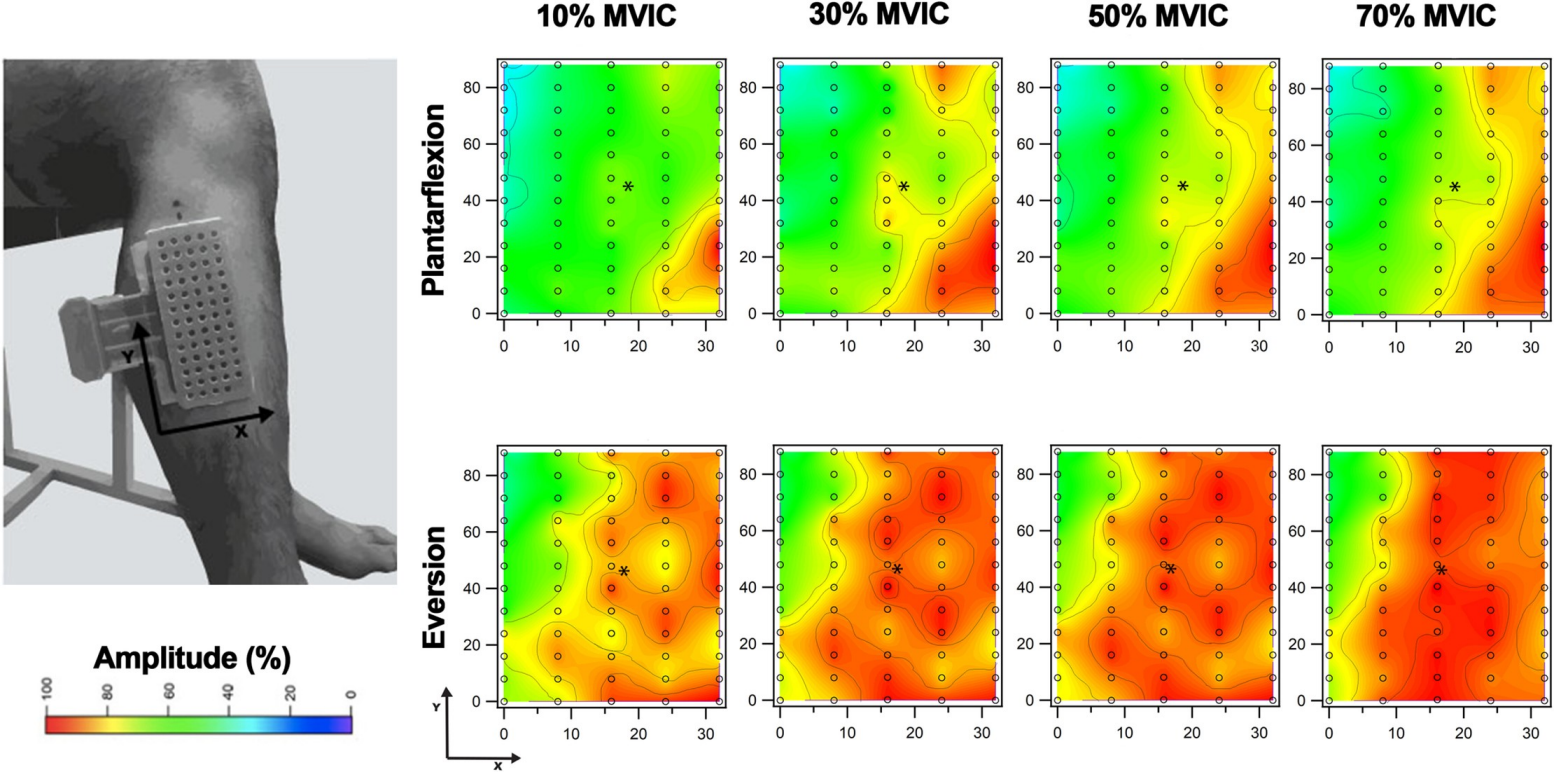
Example of the electrode grid and topographic maps of the peroneus longus electromyographic activity. Surface electrodes arranged in 5 columns which represented the anterior (columns 1/2) and posterior (columns 4/5) compartments. The electromyographic recording of the electrodes in column 3 was obtained to represent the muscle electrical activity topographically, but it was not used for the statistical analysis. In addition, the figure shows examples of topographic maps of the amplitude of the electromyographic activity recorded during 10, 30, 50 and 70% of maximum voluntary isometric contraction (MVIC). The x and y-axis values are represented in millimeters.

Neuromuscular activation of the peroneus longus muscle was assessed by multi-channel sEMG. First, a researcher shaved the skin on the anterolateral region of the dominant leg, then cleaned it with an abrasive paste (Everi, Spes Medica, Italy) and subsequently washed it with water. Then, the researcher drew a reference line between the top of the head and the lateral malleolus of the fibula. This line was considered the anatomical landmark frame (ALF). To check the correct orientation of the fibers of the peroneus longus muscle, the direction of propagation of the action potentials was evaluated with a linear electrode array (inter-electrode distance of 2.5 mm, SA 16/5, Bioelettronica, Torino, Italy) where the center of this was placed after 32% of the ALF [21, 31]. Then, the participant performed an isometric contraction of plantarflexion and eversion, equivalent to 10% of the MVIC. The participant received visual feedback of the torque record from a monitor placed in front of him. Once the correct orientation of the peroneus longus muscle fibers had been confirmed, a semi-disposable adhesive grid of 64 electrodes (ELSCH064R3S, OT Bioelecttronica, Torino, Italy) was made of 13 rows and five columns of electrodes (1 mm diameter, 8 mm inter-electrode distance) with one missing electrode at the upper corner (**Fig 2**).

In this way, a grid of 64 electrodes was configured, and conductive gel was inserted into its cavities. The center of the adhesive electrode grid was positioned over 32% of the ALF based on the previous step. The columns of the electrode grid represented two superficial NMCs of the peroneus longus muscle according to the following organization: columns 1/2 (anterior compartment), column 3 (borderline region), and column 4/5 (posterior compartment). The sEMG recording of the electrodes in column 3 was obtained with the purpose of representing the muscular electrical activity topographically, but it was not used for statistical analysis. Previous research has shown that the peroneus longus muscle belly, composed of the anterior and posterior superficial compartments, has a width of 35 mm and a length of 12.4 cm [19] in close contact with the skin, which meets the recommendations for placing an electrode grid with a lower probability of crosstalk between neighboring muscles [32]. For these reasons, a width (anteroposterior direction) of 32 mm was considered for the configuration of the electrode grid (five electrodes in line: 8 mm inter-electrode distance).

Monopolar sEMG signals were recorded at a sampling frequency of 2048 Hz, amplified by a factor of 200, and filtered with an 8th order Bessel bandpass filter at 10–500 Hz (anti-aliasing filter) and converted to digital form by a 12-bit analog-to-digital converter (EMG-USB2, Bioelettronica, Torino, Italy). The signals of the torque sensor were amplified and digitalized as an auxiliary channel, using the same device used for the sEMG signals (EMG-USB2, Bioelettronica, Torino, Italy). All signals were recorded and stored using data acquisition software (OT Biolab V1.7, Bioelettronica, Torino, Italy). Finally, the sEMG activity was recorded during the execution of the submaximal isometric voluntary contraction protocol at 10%, 30%, 50%, and 70% MVIC for each plantarflexion and eversion movement. Volunteers performed three repetitions for each percentage of MVIC in a random order. Each repetition lasted nine seconds, with a two-minute rest period between trials. Participants were allowed to practice the tests for one time in each submaximal contraction for familiarization and they received real-time visual feedback of the force with a bar graph displayed on a monitor in front of them to help control its magnitude.

### sEMG signal processing

Record monopolar sEMG signals were off-line filtered with a second-order Butterworth digital filter and a bandwidth of 20–400 Hz (OT BioLab V1.7, OTBioelettronica, Turin, Italy). The 64 monopolar signals were differentiated in the cephalocaudal direction of the matrix, thus, resulted fifty-nine simple differential signals. The processing of the sEMG signals of plantarflexion and eversion movements considered two stages: i) the amplitude of sEMG signals was calculated using RMS. A 500 ms window without overlap was used for the signals recorded during submaximal voluntary isometric contractions, and a 50 ms window for the signals recorded during the MVIC. The MVIC was determined for each of the recorded signals, and later the normalization of the sEMG amplitude of each signal obtained during the protocols for 10%, 30%, 50%, and 70% MVIC was obtained. Only the central five seconds of the nine seconds recorded were considered for the analysis in order to reduce fluctuations in the first two seconds and the effect of fatigue during the last two seconds [4, 25]. At each level of submaximal voluntary isometric contraction and its central five seconds of the sEMG signals, ten normalized and subsequently averaged sEMG amplitude values were obtained. Furthermore, this average value was calculated in each column (i.e., NMC) of the electrode grid; ii) the topographic distribution of the sEMG amplitude—at each level of submaximal isometric voluntary contraction and ankle movement—was described through the COM position in the anteroposterior (COMx) and cephalocaudal (COMy) components in relation to the position of the electrode grid [4]. For this, the COMx and COMy were calculated with the RMS of the fifty-nine electrodes. Topographic maps were constructed to describe the distribution of the sEMG amplitude of the NMCs at each level of submaximal isometric voluntary contraction and ankle movement (**Fig 2**). The maps were constructed with the RMS values and the position coordinates of each of the fifty-nine electrodes. These data were 2D interpolated used a smoothing spline model and eight-factor smoothing [4]. All of the above procedures were performed with IgorPro 6.0 software (WaveMetrics Inc, Portland, USA).

### Statistical analysis

A descriptive statistical analysis (mean and standard deviation) was calculated for the sEMG amplitude (expressed as MVIC percentage: 10%, 30%, 50% and 70%), COMx and COMy in each compartment (columns 1/2 and 4/5) and ankle movement (eversion and plantarflexion). The distribution of normality and homogeneity of variance were evaluated through the Shapiro-Wilk test and the Levene test, respectively. To determine possible interactions between EMG amplitudes and ankle movements, a two-way ANOVA (EMG amplitude x movement) was applied for COMx and COMy. Furthermore, to determine possible interactions between compartments (columns) of the peroneus longus and ankle movement, a two-way ANOVA (column x movement) was applied for each EMG amplitude. In the case of interactions, a post-hoc analysis with Tukey’s multiple comparison test was performed. For all analyzes, an alpha of 0.05 was considered and GraphPad Prism version 8.0.0 for Mac software was used (GraphPad Software, San Diego, California USA).

## Results

All participants (twenty-two volunteers) were included in the analysis. The data presented a normal distribution and homogeneity of variance, and they were expressed as means and standard deviations (**Table 1**).

**Table 1:** Descriptive statistics.

**Eversion**	**10% MVIC**	**30% MVIC**	**50% MVIC**	**70% MVIC**
COMx	16.9 ± 1.3	16.9 ± 1.4	17.0 ± 1.5	16.7 ± 1.2
COMy	47.6 ± 2.2	47.3 ± 2.3	47.5 ± 2.2	47.1 ± 1.9
Columns 4/5	58.4 ± 15.7	58.7 ± 13.8	57.3 ± 13.1	59.1 ± 11.9
Columns 1/2	61.8 ± 10.0	62.9 ± 10.5	62.0 ± 10.8	61.3 ± 11.4
**Plantarflexion**	**10% MVIC**	**30% MVIC**	**50% MVIC**	**70% MVIC**
COMx	18.9 ± 1.8	20.0 ± 1.7	19.9 ± 1.9	20.0 ± 1.8
COMy	47.7 ± 2.5	47.7 ± 2.2	48.2 ± 2.5	48.5 ± 2.5
Columns 4/5	41.3 ± 14.4	33.7 ± 11.8	32.8 ± 10.8	33.3 ± 14.4
Columns 1/2	56.8 ± 8.7	54.2 ± 7.2	51.7 ± 6.3	52.7 ± 9.4

Means and standard deviation of center of mass in anteroposterior position (COMx), center of mass in cephalocaudal position (COMy), and normalized electromyographic amplitude of peroneus longus muscle compartments during the ankle movement at different percentages of maximum voluntary isometric contraction.

MVIC, maximum voluntary isometric contraction; COMx, center of mass in anteroposterior position; COMy, center of mass in cephalocaudal position.

### COMy and COMx

**Fig 3:**
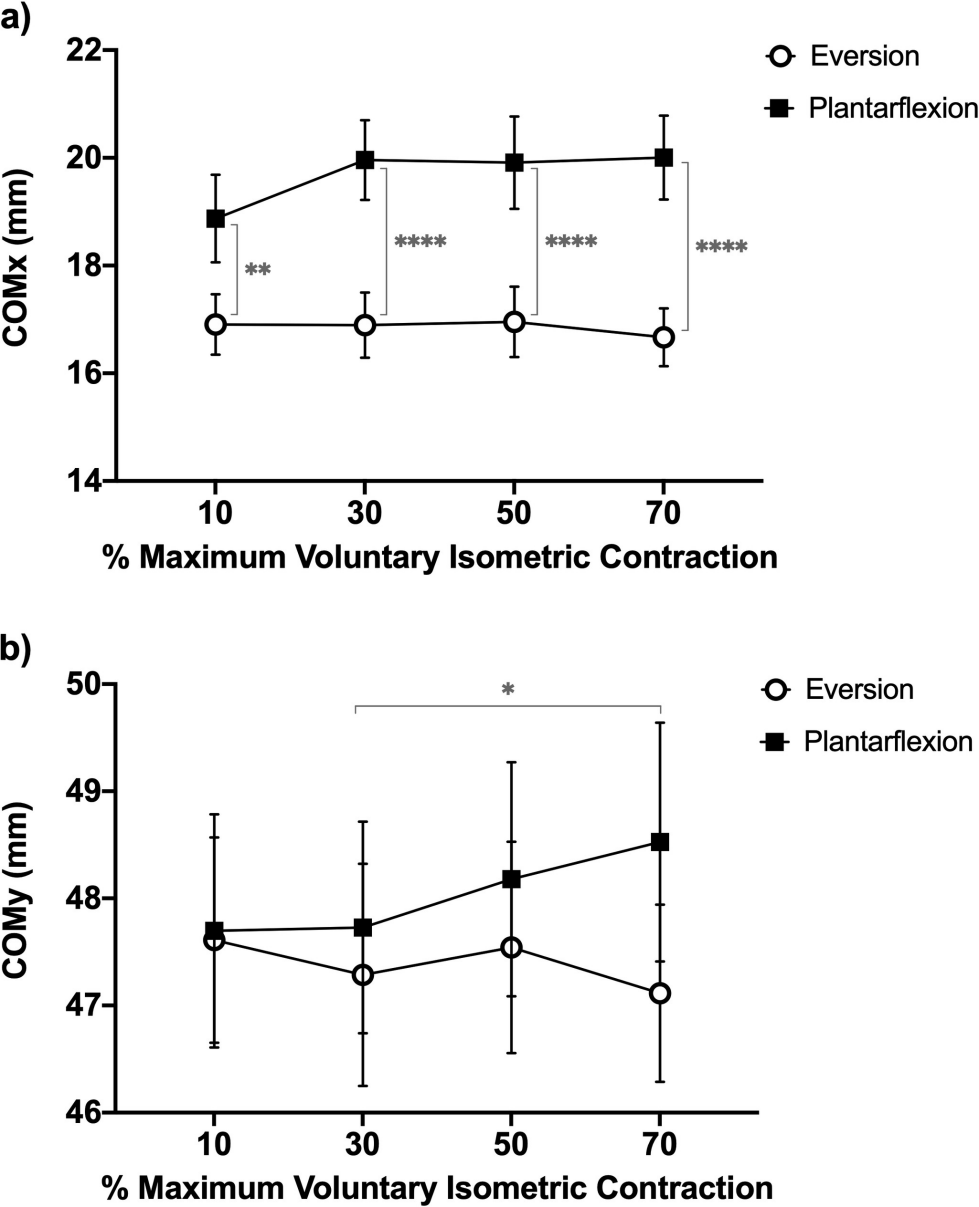
Position of the center of mass at different percentage of maximum voluntary isometric contraction. a) Position of the center of mass in X. b) Position of the center of mass in Y. The significant differences are indicated by clapped line with *p*-values according to the following symbology: * = *p* < 0.05; ** = *p* < 0.01; **** = *p* < 0.0001.

The ANOVA revealed a significant sEMG amplitude x movement interaction (*F*_1.97, 41.41_ = 6.127; *p* = 0.0048) for COMx position. Post-hoc analysis showed that COMx during eversion was significantly (*p* < 0.01) displaced to the posterior part of the peroneus longus compared to the position of COMx during plantarflexion in all MVIC percentages (**Fig 3**).

In addition, the ANOVA revealed a significant sEMG amplitude x movement interaction (*F*_1.95, 41.08_ = 3.541; *p* = 0.0391) for COMy position. Post-hoc analysis showed that COMy during plantarflexion at 70% MVIC was significantly (*p* = 0.0260) displaced to the cephalic part of the peroneus longus compared to the position of COMy during plantarflexion at 30% MVIC (**Fig 3**). However, no significant COMy differences were observed between movements (eversion and plantarflexion) in each MVIC.

### Movements and compartments

**Fig 4:**
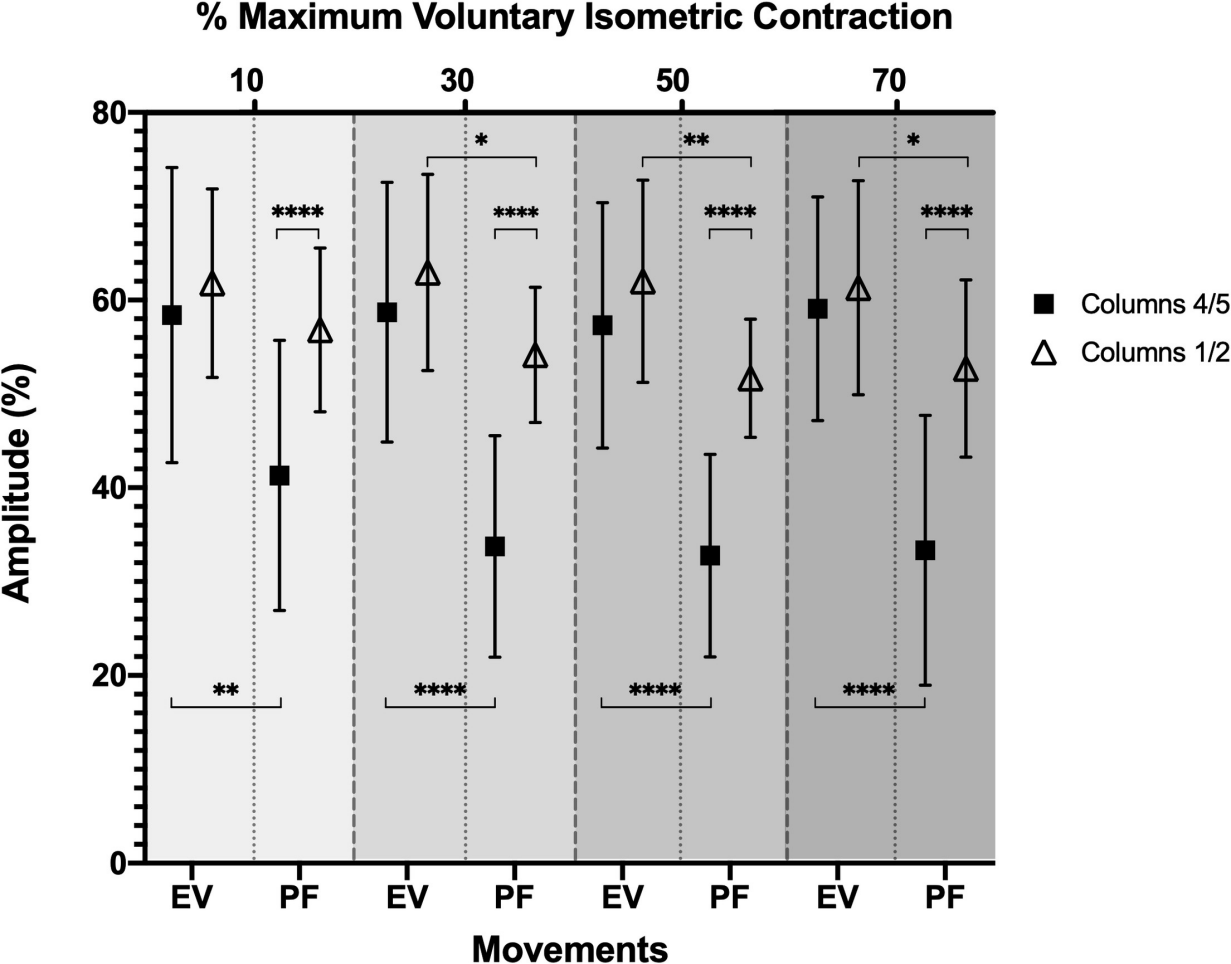
Surface electromyographic amplitude (mean and standard deviation) of each column (1/2 and 4/5) of the peroneus longus muscle during eversion and plantarflexion movements at 10, 30, 50 and 70% of the maximum voluntary isometric contraction. EV, eversion; PF, plantarflexion. The significant differences are indicated by clapped line with *p*-values according to the following symbology: * = *p* < 0.05; ** = *p* < 0.01; **** = *p* < 0.0001.

The ANOVA showed a significant columns x movement interaction for sEMG amplitude at 10% (*F*_1.00, 21.00_ = 20.80; *p* = 0.0002), 30% (*F*_1.00, 21.00_ = 57.94; *p* < 0.0001), 50% (*F*_1.00, 21.00_ = 32.94; *p* = 0.0001), and 70% MVIC (*F*_1.00, 21.00_ = 60.50; *p* = 0.0001). Post-hoc analysis revealed that the sEMG amplitude of columns 1/2 was significantly higher (*p* < 0.0001) than the columns 4/5 in all MVIC percentages of plantarflexion movement (**Fig 4**).

Conversely, there were no significant sEMG amplitude differences (*p* > 0.05) between columns in each MVIC percentages of eversion movement (**Fig 4**). On the other hand, the post-hoc analysis for columns 1/2 showed that the sEMG amplitude of eversion was significantly higher than the plantarflexion at 30% MVIC (*p* = 0.0144), 50% CVIM (*p* = 0.0019), and 70% MVIC (*p* = 0.0110), although no significant sEMG amplitude difference (*p* > 0.05) was observed between columns at 10% MVIC. Finally, the post-hoc analysis for columns 4/5 showed that the sEMG amplitude of eversion was significantly higher than the plantarflexion in each MVIC percentages (**Fig 4**).

## Discussion

The primary finding of this study was that eversion showed significantly higher sEMG amplitude than plantarflexion in columns 4/5 (posterior compartment) at low, moderate, and high percentages of MVIC. However, no significant difference in sEMG amplitude was observed in column 1/2 between eversion and plantarflexion. Therefore, the hypothesis formulated in the present investigation is partially accepted: the peroneus longus muscle presented NMCs compatible with a spatial distribution depending on the motor task; the anterior compartment contributed to both eversion and plantarflexion movements, whereas the posterior compartment mainly contributed to the eversion movement of the ankle in low, moderate, and high percentages of MVIC.

Our results indicated a posterior displacement of the COMx in the eversion movement compared to the plantarflexion movement of the ankle (*p* < 0.0001) at all MVIC percentages, with greater topographic distancing of the COMx at higher levels of activation (**Fig 3**). This shows a higher recruitment of motor units contributing to the sEMG detected by columns 4/5 (posterior compartment) of the peroneus longus muscle during eversion. This initial result was verified by studying the RMS between the compartments in low, moderate, and high percentages of MVIC (**Fig 4**). The differences observed in low to high intensities of contraction have been observed in other human muscles. Gao et al. [10] investigated the spatial organization of the extensor digitorum communis during force production at 15%, 30%, and 45% MVIC, observing significant differences between the regions of the common extensor digitorum muscle at all contraction intensities. Otherwise, Guzmán-Venegas et al. [4] only observed greater sEMG activation of the anterior compartment compared to the posterior compartment of the superficial masseter muscle at low (20% MVIC) and moderate (60% MVIC) contraction intensities during an isometric bite with a constant interocclusal distance. The authors attributed these differences to the mechanical advantage of the anterior compartment and, therefore, a lower need to recruit its motor units. On the contrary, previous reports have only observed significant differences in RMS at moderate and high intensities of contraction between regions (proximal and distal) of the rectus femoris muscle [3]. Watanabe et al. [3] attributed these observations to the non-linear relationship between sEMG amplitude and force production, which could reflect the various motor unit recruitment strategies and the different types of motor units distributed spatially in a non-homogeneous way in the muscle. Although, in our investigation, the non-linear behavior between the amplitude sEMG and the force produced was not observed, a smaller difference was shown at lower intensities (10% MVIC) compared to higher intensities (70% MVIC) in the movement of plantarflexion of the ankle. It is possible that this non-linear behavior could have been observed at very low contraction intensities (<10% MVIC), and the linear or non-linear sEMG/force relationship depends on the anatomic, biomechanical, and neurophysiological characteristics of human muscles. In this context, Woods & Bigland-Ritchie [33] attributed this diversity of behavior to the varied fiber composition, fiber distribution, and force-generating patterns. They suggested a linear relationship for muscles of near-uniform fiber composition and a non-linear relationship for muscles of mixed fiber composition. For these reasons, it is necessary to develop future research that attempts to elucidate the mechanisms that explain the various observations of regional activation at different levels of contraction.

Previous research has reported that the peroneus longus muscle shows greater activation during eversion, as its eversion lever arm is 2.0 times longer than its plantar flexion lever arm [17]. In this context, our results showed that eversion revealed significantly higher RMS values than plantarflexion in columns 4/5 (posterior compartment; **Fig 4**). Furthermore, ankle eversion did not show significant differences in RMS values between columns, suggesting a similar level of contribution from both NMCs and a high proportion of active motor units along the peroneus longus muscle. On the other hand, lower RMS values were observed in columns 4/5 compared to columns 1/2 in the plantarflexion movement, showing differences in contribution between compartments and a lower proportion of motor units that participated in plantarflexion. These results support the idea that muscles fulfill a region-specific functional role, i.e., the NMCs have a behavior dependent on the motor task or movement [1–3, 9, 34].

Several investigations indicate that peroneus longus NMCs have a longitudinal topographic distribution respecting a muscular bipennate conformation, each one with one or two motor points distributed within the proximal third of the muscle [6, 7, 35]. From this point of view, it is possible to estimate that the location of the center of the electrode grid is within the proximal third and covering the anterior and posterior compartments. Based on this anatomical organization, the installation of the electrode grid makes it possible to acquire the sEMG signals, and in this way, to assume that the anterior compartment mainly contributes to plantarflexion and the posterior compartment to eversion of the foot in a non-weight bearing position. On the other hand, it has been observed that the stimulation of the motor points of the posterior compartment produces the contraction of its fibers and the subsequent articular movement of eversion in a non-weight bearing position evaluated through triaxial accelerometry [5]. Based on the above, both the anatomical and biomechanical organization of the posterior compartment of the peroneus longus muscle are consistent with our electrophysiological results measured with multi-channel sEMG. On the other hand, it has been observed that the stimulation of the motor points of the anterior compartment generates the dorsiflexion movement of the foot in a non-weight bearing position [5], although in our investigation, the activation of the anterior compartment was observed during plantarflexion (**Fig 4**). These differences can be explained due to the weight bearing or non-weight bearing position of the foot since biomechanical studies have shown that the peroneus longus muscle participates in the plantarflexion of the ankle to maintain the longitudinal arch of the foot during the stance (weight bearing) phases of gait and running [16–18]. However, in non-weight bearing positions of the foot, e.g., the terminal swing phase of the gait cycle, activation of the peroneus longus muscle contributes synergistically to the dorsiflexion movement to control the rate of inversion occurring at the ankle joint [36].

The present study has some limitations that are necessary to recognize: i) the inter-subject variability related to the length of the leg and the peroneus longus muscle may have influenced the anatomical landmarks and therefore the correct installation of the electrodes grid and sEMG amplitude measurements [21]; ii) sEMG is susceptible to crosstalk. sEMG activity measured from the anterior and posterior neuromuscular compartments could have been contaminated by the close sEMG activity of the tibialis anterior and lateral gastrocnemius. In addition, low intensity MVIC (10%) may generate a high amplitude EMG of the target muscle. This may be due to the difficulty of voluntarily and differently recruiting the peroneous longus muscle at a low intensity of MVIC (10%) during eversion (primary action) or plantar flexion (secondary action), which can lead to recruitment of motor units nearby muscles, such as plantar flexors, in order to achieve the required motor task. This may have occurred despite the familiarization and learning protocol described in the procedures section of the present investigation; and iii) a deep central tendon has been observed between the anterior and posterior compartments [6], which could affect the quality of the sEMG signals, due to the noise that the tendon could cause and the possible end-of-fiber effect [37, 38]. However, in an effort to reduce these factors, our research relied on previous investigations where the correct location of the electrodes grid was determined according to a leg length measurement protocol, i.e., ALF, and the optimal position of the electrode from the origin of the ALF [21, 31]. Furthermore, an interelectrode distance of 8 mm was used, considered an adequate distance to reduce the crosstalk contamination on leg muscles [31, 39]. As commend in the *Methods and materials* section, it has been reported that the peroneus longus muscle has a width of 35 mm, which allows the correct acquisition of a sEMG signal and thus reduces the probability of crosstalk of neighboring muscles [32]. All these considerations allowed an adequate location of the electrodes, fulfilling various criteria regarding sEMG signal quality, region free from innervation zones, visual detectability of the action potentials propagation, and detectability of motor units [31].

The results of the present investigation demonstrated a spatial topographic distribution, i.e., NMCs, in the peroneus longus muscle during the execution of the eversion and plantarflexion movements of the ankle. These results may contribute to updating the model of chronic ankle instability [22–24]. This considers that motor impairments such as muscle weakness, muscle arthrogenic inhibition, and delayed muscle reaction times are one of the main elements of the model that help explain the development of this dysfunction [22–24]. The evaluation of these motor impairments from a compartmentalization perspective could help in the detailed identification of alterations in one or more compartments of the peroneus longus muscle. It has been observed that patients with chronic ankle instability present a decrease in the isometric, concentric and eccentric strength of the eversors muscles [22, 40]. According to our results, the posterior compartment contributes mainly to the eversion movement of the ankle, therefore it could be the compartment most affected in this type of dysfunction. Future studies should use multi-channel sEMG to evaluate regional differences in other muscles, as well as to identify possible differences in compartment activation when comparing exercises prescribed to increase stability in individuals with chronic ankle instability. This knowledge will help to identify the most suitable therapeutic exercises and improve the neuromuscular function following an ankle injury.

In conclusion, the present study investigated the contribution of peroneus longus NMCs to eversion and plantarflexion movements through multi-channel sEMG. The peroneus longus muscle presented NMCs compatible with a functional spatial distribution depending on the motor task; specifically, the anterior compartment contributed to both eversion and plantarflexion movements, whereas the posterior compartment mainly contributed to the eversion movement of the ankle in low, moderate, and high percentages of MVIC.

## Supporting information

S1 File Data set. (XLSX)
